# The Efficacy and Safety of Bumetanide in Children with Autism Spectrum Disorder: An Updated Meta-analysis

**DOI:** 10.1007/s00787-025-02890-8

**Published:** 2025-10-30

**Authors:** Nada Ibrahim Hendi, Nereen A. Almosilhy, Omar Hany Mohammed, Amro Mamdouh Abdelrehim, Ahmed Eisa, Heba-tullah Ahmed Abouelkassem Saddoun, Abdelbassat Belakhdar

**Affiliations:** 1 Faculty of Medicine, Ain Shams University, Cairo, Egypt; 2 Department of Pharmacology and Toxicology, Faculty of Pharmacy, Tanta University, Tanta, Egypt; 3 Faculty of Medicine, Zagazig University, Zagazig, Egypt; 4 Faculty of Medicine, Misr University for Science and Technology, 6th of October, Giza, Egypt; 5 Faculty of Medicine, Al-Azhar University, Cairo, Egypt; 6 Department of Psychiatry, Faculty of Medicine, University of Oran1 Ahmed Ben Bella, Oran, Algeria

**Keywords:** Autism, ASD, Bumetanide, Diuretic

## Abstract

Autism spectrum disorder (ASD) is a complex range of neurodevelopmental disorders. The treatment of ASD is challenging since there are no approved drugs except for risperidone and aripiprazole, which have a limited effect on core symptoms and are associated with a wide range of adverse events. Bumetanide is a loop diuretic suggested to play a role in ASD symptoms by modulating the GABAergic system. This meta-analysis aims to evaluate the efficacy and safety of bumetanide on ASD symptom management. We included randomized controlled trials comparing bumetanide with placebo or conventional treatment. Our primary outcomes included the Childhood Autism Rating Scale (CARS), Social Responsiveness Scale (SRS-2), Clinical Global Impression- Efficacy Index (CGI-EI), Social interaction (SI), repetitive behavior and restricted interests (RRB), and adverse events. Bumetanide was associated with significant improvement in CARS total score (MD = -2.28; 95% CI = [-4.07, -0.49], *p* = 0.01) and CGI-EI (MD = 0.27; 95% CI = [0.09, 0.44], *p* = 0.003) compared to placebo. However, no significant difference was found in the SRS-2 score. Findings for SI and RRB were inconsistent depending on the pooled scales, with significant results observed when pooling SRS-2 but not when pooling ADOS. Bumetanide was generally safe and well-tolerated. Common adverse events include polyurea, hypokalemia, and dehydration. Although statistically significant improvements were observed on some measures, we cannot conclude the superiority of bumetanide in alleviating ASD symptoms. Further large clinical trials should be conducted to determine the effect of bumetanide on different symptoms of ASD and the variation in treatment effect among different patient populations.

**Supplementary Information:**

The online version contains supplementary material available at 10.1007/s00787-025-02890-8.

## 1. Introduction

Autism spectrum disorder (ASD) is a group of neurodevelopmental disorders characterized by varying degrees of impairment in social interaction, repetitive behavior, and restricted interest [1]. Treatment of ASD is mainly behavioral and speech therapy. Medications can be used as adjunct therapy for behavioral issues. Since it is a complex spectrum of disorders, it’s difficult to develop specific treatment against the core symptoms of ASD [2]. The only approved symptomatic interventions for ASD are risperidone and aripiprazole. However, they only target irritability and aggression, not the core symptoms such as repetitive behaviors or social interactions. While some behavioral improvement can occur, these drugs cause a wide range of adverse effects, such as sedation, vomiting, weight gain, increased appetite, and extrapyramidal symptoms [3, 4]. Thus, it becomes essential to identify and assess other effective therapeutic options with fewer adverse effects.

Bumetanide is a loop diuretic and a selective inhibitor of Na-K-Cl cotransporter 1 (NKCC1) [5]. Experimental studies suggested that bumetanide can improve autistic symptoms by reducing the intracellular chloride, leading to GABAergic inhibition, which is found to play a role in ASD symptoms [6]. Previous meta-analysis reported supportive data for the use of bumetanide in ASD patients [7]. However, some statistical flaws were identified in this meta-analysis such as duplicate sample size and inappropriate analysis model. Additionally, there are conflicting results with the more recent clinical trials. Two large phase III randomized controlled trials, which included a sample size almost equal to that included in the previous meta-analysis, didn’t show a superior effect of bumetanide in ASD patients [8]. Thus, we conducted this updated meta-analysis to re-evaluate and provide updated evidence regarding the efficacy of bumetanide in ASD patients.

## 2. Methods

This updated systematic review, and meta-analysis was conducted with strict adherence to the Preferred Reporting Items for Systematic Review and Meta-Analysis (PRISMA) statement guidelines [9]. Our study protocol was prospectively registered in the International Prospective Register of Systematic Reviews (PROSPERO), (registration number: CRD42024562086).

### 2.1. Literature search strategy

We searched the following medical electronic databases: PubMed, Web of Science, and Scopus from inception till June 24, 2024. No restrictions or filters were applied. Our final search strategy included the following search terms: (“autism” OR “Autistic*” OR “ASD”) AND (“bumetanide”). Other terms such as “Kanner Syndrome”, “Asperger syndrome”, “Pervasive Developmental Disorder-Not Otherwise Specified”, “PDD-NOS”, “Bumex”, “Drenural”, “Miccil”, “Bumedyl”, “Burinex” were excluded as they did not make a difference in the number of retrieved results.

Duplicate records were removed using EndNote 9 software. A manual screening of references was performed in the included studies to identify additional studies.

### 2.2. Eligibility criteria and study selection

Two independent authors performed screening for eligible studies using Rayyan software [10] in two consecutive steps: title and abstract screening, followed by full-text screening. The studies that met the inclusion criteria or had insufficient abstract information were proceeded to the full text screening. Potentially eligible studies were obtained and independently evaluated by two reviewers, using the predefined inclusion criteria. Disagreements were resolved by consensus or by referring to the first author. We included studies with the following criteria: (1) Population: children aged less than 18 years with an expert confirmed ASD diagnosis according to the DSM-V or ICD-10, (2) Intervention: Bumetanide diuretic with no restrictions on the dose, frequency, or treatment duration, (3) Comparison: Placebo or conventional treatment, (4) Outcome: at least one outcome measurement of the core symptoms of ASD or safety measurements, and (5) Study design: randomized controlled trials. Observational studies, editorials, letters, book chapters, conference papers, case reports, reviews, single-arm studies, and research published in languages other than English were excluded from our study.

### 2.3. Data collection and risk of bias assessment

Two independent authors performed data extraction using a predefined data extraction sheet, which contains the following domains: study setting and summary, demographic variables including participants’ age and sex, Data about the intervention: duration and dosage of bumetanide, outcome measurement, and adverse effects. Any conflicts were resolved by consensus or referral to the first author.

Our outcomes of interest were Social Responsiveness Scale 2 (SRS-2), Childhood Autism Rating Scale (CARS), Clinical Global Impression-Efficacy Index Scale (CGI-EI), social interaction, repetitive behavior, and sensory affection. They were extracted as means and standard deviations. We used Intention-to-treat (ITT) data whenever available. For Dai, Du, and Fuentes, ITT was not reported, and we used per-protocol values instead. The number lost to follow-up was minor, and we believe it doesn’t impact the results of our study.

Graphical data were obtained using the web plot digitizer software (Plot Digitizer, version 2.6.8, Free Software Foundation, Boston, MA, USA).

### 2.4. Quality assessment

Two independent authors assessed the quality of the included studies using the Cochrane risk of bias assessment tool for RCTs version two (ROB2) [11]. We assessed bias in the following domains: the randomization process, deviations from intended interventions, missing outcome data, measurement of the outcome, and selection of the reported result. The overall authors’ judgment for each domain fell into one of three categories: low, some concerns, or high risk of bias. Conflicts were resolved by consensus or referral to the first author.

### 2.5. Measures of treatment effect and data synthesis

For SRS, CGI, and CARS, data were pooled as Mean difference (MD) and 95% confidence interval (CI). However, for social interaction, repetitive behavior, and sensory affection, data were pooled as standardized mean difference (SMD) and 95% CI due to the variation in the scale used to assess these symptoms. All analyses were performed RevMan software using random effect model.

### 2.6. Subgroup analysis

We stratified the included studies into subgroup analysis based on the dose and assessment timing, and assessment tool to investigate their effect on treatment response.

### 2.7. Assessment of heterogeneity

Heterogeneity was assessed by visual inspection of the forest plots and measured using the Chi-Square test and *I*-square test. A p-value of less than 0.1 for the Chi-square test was considered significant for heterogeneity. Whereas the *I*-square test was used to measure the magnitude of heterogeneity. In case of significant heterogeneity, we performed a sensitivity analysis (leave one out analysis) to assess the effect of single study removal on the overall effect size.

### 2.8. Assessment of publication bias

In agreement with Egger et al., because the number of included studies was less than 10, it was inapplicable to examine potential publication bias via Egger’s test for the funnel plot asymmetry [12].

## 3. Results

### 3.1. Description of the included studies

**Fig. 1:**
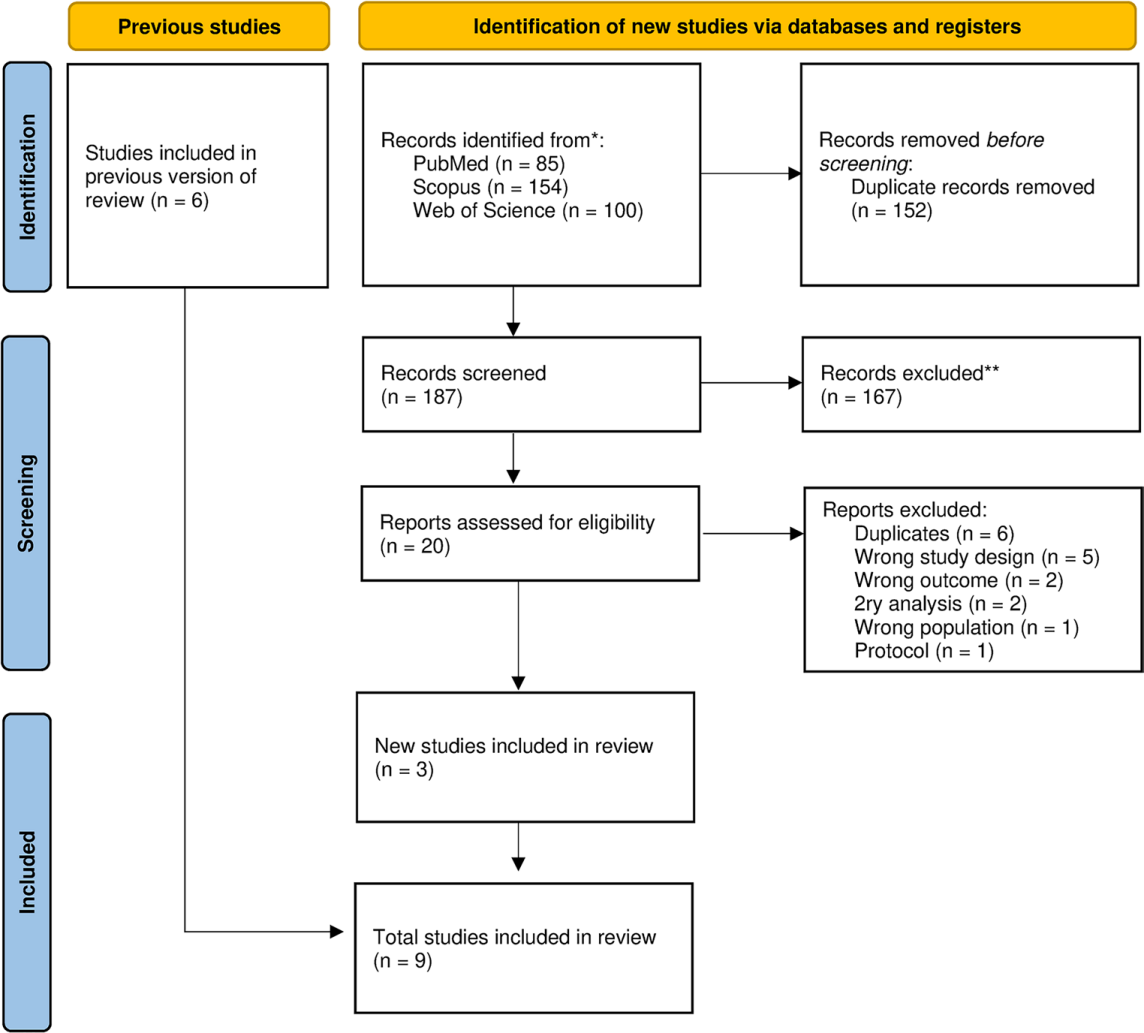
PRISMA flowchart

Our literature search retrieved a total of 339 articles from different online databases. 152 duplicates were removed. We then excluded 167 records in the title and abstract screening. The remaining studies underwent full-text screening, after which we included 9 RCTs from 8 publications in our systematic review and meta-analysis. An updated search on June 26, 2024, was done, and didn’t retrieve any additional studies. A detailed description of the selection process is shown in the PRISMA flowchart (Fig. 1).

We included a total of 9 RCTs comprising 920 patients who had a diagnosis of autism spectrum disorder according to the Diagnostic and Statistical Manual-5th edition (DSM-5) criteria, except Lemonnier 2012 [13], which followed the 10th revision of the International Statistical Classification of Diseases and Related Health Problems (ICD-10) criteria. Fuentes 2023 consisted of two RCTs, which were referred to as Fuentes (A) 2023 and Fuentes (B) 2023 [8]. A detailed description of the summary characteristics of the included studies is shown in Table 1.

**Table 1:** Summary and characteristics of the included studies

Study ID	Age	Male/Female *n* (%)	Exclusion Criteria	Intervention dose and duration	Adverse Events Intervention	Adverse Events Control
Dai 2021 China *n* = 119	3–6 years	51/8	Liver/kidney diseases Allergy to sulfa drugs Abnormal ECG Genetic/chromosomal abnormality Other neurological/psychiatric disorders Severe hearing/visual impairment Currently using melatonin/cessation for < 3 weeks	0.5 mg twice daily for 3 months	Polyuria 40 (67.8%) Loss of Appetite 4 (6.8%) Constipation 5 (8.5%) Nausea 1 (1.7%), Vomiting 1 (1.7%), Diarrhea 0 (0%) Sleeping problem 1 (1.7%) Mild hypokalemia 5 (8.5%) Mild hyperuricemia 2 (3.4%) Serious adverse events 0 (0%)	Polyuria 5 (8.3%) Loss of Appetite 1 (1.7%) Constipation 2 (3.3%) Nausea 0 (0%) Vomiting 2 (3.3%), Diarrhea 1 (1.7%), Sleeping problem 0 (0%) Mild hypokalemia 0 (0%) Mild hyperuricemia 1 (1.7%) Serious adverse events 0 (0%)
		49/11				
Du 2015 China *n* = 55	2.5–6.5 years	27/5	Liver/kidney diseases Abnormal ECG Genetic/chromosomal abnormality Congenital metabolic disorders Change in routine urine/blood tests, electrolytes, and glucose tests. History of seizures	0.5 mg twice daily for 3 months	No serious adverse events	
		24/4				
Lemonnier 2012 France *n* = 54	3–11 years	24/3	Liver/kidney diseases Chromosomal abnormality Allergy to sulfa drugs Abnormal ECG Other neurological disorders	0.5/1/2 mg twice daily for 3 months	Bed-wetting 1 (3.7%) Hypokalemia 6 (22.2%)	Bed-wetting 1 (3.7%) Eczema 1 (3.7%)
		20/7				
Lemonnier 2017 France *n* = 88	2–18 years	16/4	Epilepsy use of psychotropic medications (antipsychotic, psychostimulant, antidepressant, anxiolytics, mood stabilizers, and neuroleptic agents) or discontinuation for < 4 weeks	0.5 mg twice daily for 3 months	Hypokalemia 6 (30%)/Polyuria 2 (10%)/Asthenia 2 (10%)/Abdominal pain 1 (5%)/Polydipsia 1 (5%)	Diarrhea 3 (13.6%)
		19/4				
Sprengers 2021 Netherlands *n* = 75	7–15 years	32/15	Liver/Kidney disease Allergy to sulfa drugs IQ < 55 Unstable serious illness Psychoactive medication use < 8 weeks, except melatonin NSAIDs use CBT Neurological disorders	0.5 mg twice daily for 3 months ^**a**^	Orthostatic hypotension 17(36%) Dehydration 8(17.4%) Hypokalemia 24(51%) Hypoglycemia 1 (2%)	Orthostatic hypotension 5(11%) Dehydration 1(2.3%) Hypoglycemia 3(7%)
		31/14				
Zhang 2020 China *n* = 83	3–6 years	36/6	Currently using melatonin or its cessation for < 3 weeks Structural abnormalities in the brain MRI contraindications	0.5 mg twice daily for 3 months	Polyuria 15 (35.7%) Hypokalemia 4 (9.5%) Loss of appetite 4 (9.5%) Fatigue 1 (2.38%)	NA
		29/12				
Fuentes (A) 2023 multicenter (13 countries) *n* = 183	7–17 years	87/20	Liver/Kidney/Heart disease Genetic/chromosomal abnormality Syndromic children, like Rett syndrome/fragile X Psychiatric disorder/high suicide risk Unstable psychotherapy/CBT concomitant psychotropic medication ^**C**^	0.5 mg twice daily for 6 months ^**b**^	Thirst Polyuria Dry mouth Loss of appetite Hypokalemia Increased appetite Nasopharyngitis Pollakiuria	Thirst Polyuria Dry mouth Loss of appetite Hypokalemia Increased appetite Nasopharyngitis Pollakiuria
		87/17				
Fuentes (B) 2023 Multicenter (13 countries) *n* = 183	2–6 years	89/18		0.5 mg twice daily for 6 months ^**b**^		
		87/17				
Shaker 2024 Egypt *n* = 80	3–12 years	NA	Liver/Kidney disease Abnormal ECG Syndromic children, like Rett syndrome	0.5 mg twice daily for 6 months	Polyuria 15(37.5%)	NA

**a** 0.015 mg/kg for children < 30 kg/0.5 mg for children > 30 kg. Doses are doubled if the blood analysis showed no abnormalities

**b** 0.01 mg/kg for patients < 25 kg/0.5 mg for patients > 25 kg

**c** Aripiprazole and risperidone were allowed if a stable dose was used throughout the study. In Fuentes A, methylphenidate, atomoxetine, or guanfacine were allowed if stabilized for at least 4 weeks before inclusion in both trials. Patients with previous ineffective Bumetanide treatment for ASD were excluded

CBT = Cognitive behavioral Therapy

### 3.2. Quality assessment

**Fig. 2:**
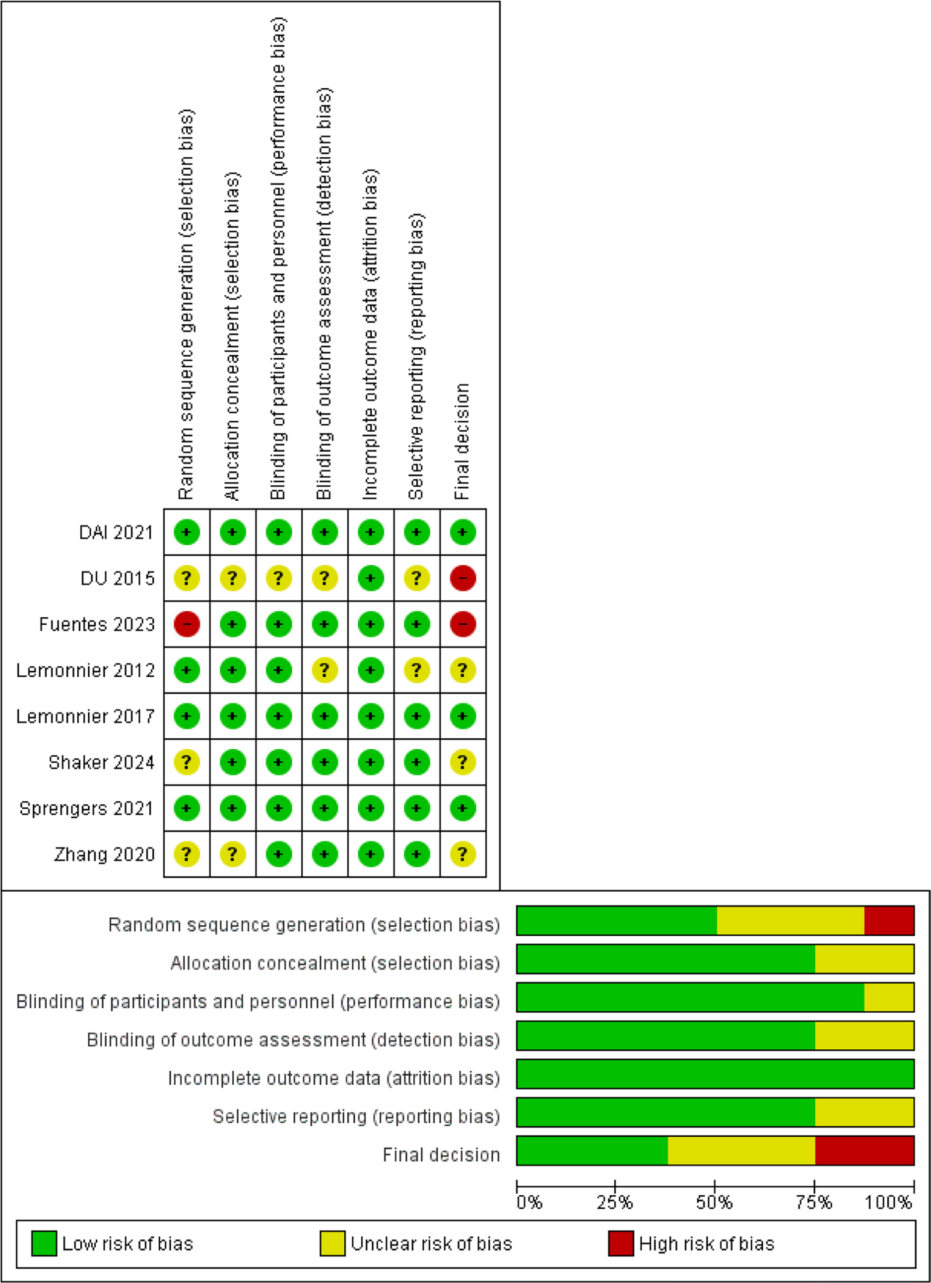
Risk of bias graph and summary of its domains

According to the Cochrane risk of bias assessment tool (ROB-2), two trials had a high risk of bias [6, 14], four had a moderate risk [8],], and two had a low risk of bias [15, 16]. The risk of bias was mainly related to blinding, random sequence generation, and allocation concealment. Risk of bias graph and summary of quality assessment domains of included studies are shown in (Fig. 2).

### 3.3. Childhood autism rating scale (CARS)

**Fig. 3:**
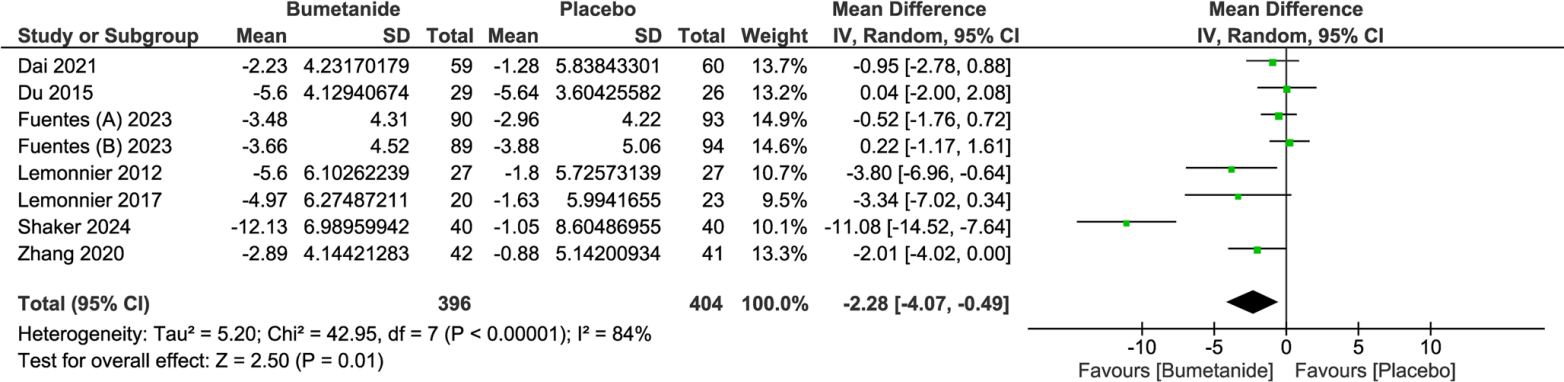
Forest plot of childhood autism rating scale (CARS) total score

The pooled analysis revealed a statistically significant difference in CARS in favor of the bumetanide group (MD = −2.28; 95% CI = [−4.07, −0.49], *p* = 0.01). This indicates significant improvements in the severity of ASD symptoms with bumetanide treatment compared to placebo. There was a significant moderate heterogeneity among the included studies (I² = 84%, *p* < 0.00001), indicating some variability in effect sizes across the included studies. (Fig. 3)

**Fig. 4:**
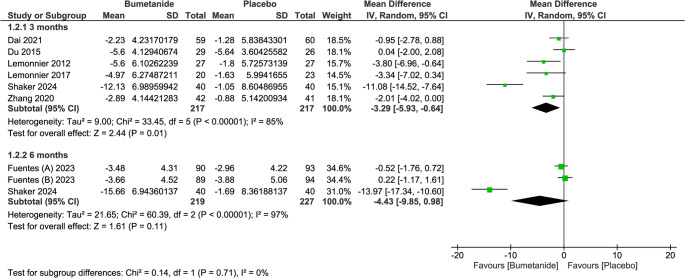
Forest plot and subgroup analysis of CARS total score based on the time of assessment

Leave-one-out analysis revealed that this heterogeneity is attributed to Shaker et al. (2024). By removing this study, the heterogeneity resolves (I2 = 38%, *p* = 0.14) without affecting the significance (MD = −0.95; 95% CI = [−1.87, −0.02], *p* = 0.04). Although subgroup analysis based on the time of assessment showed a significant difference in the 3-month subgroup only, the test for subgroup difference was not significant. (Fig. 4)

**Fig. 5:**
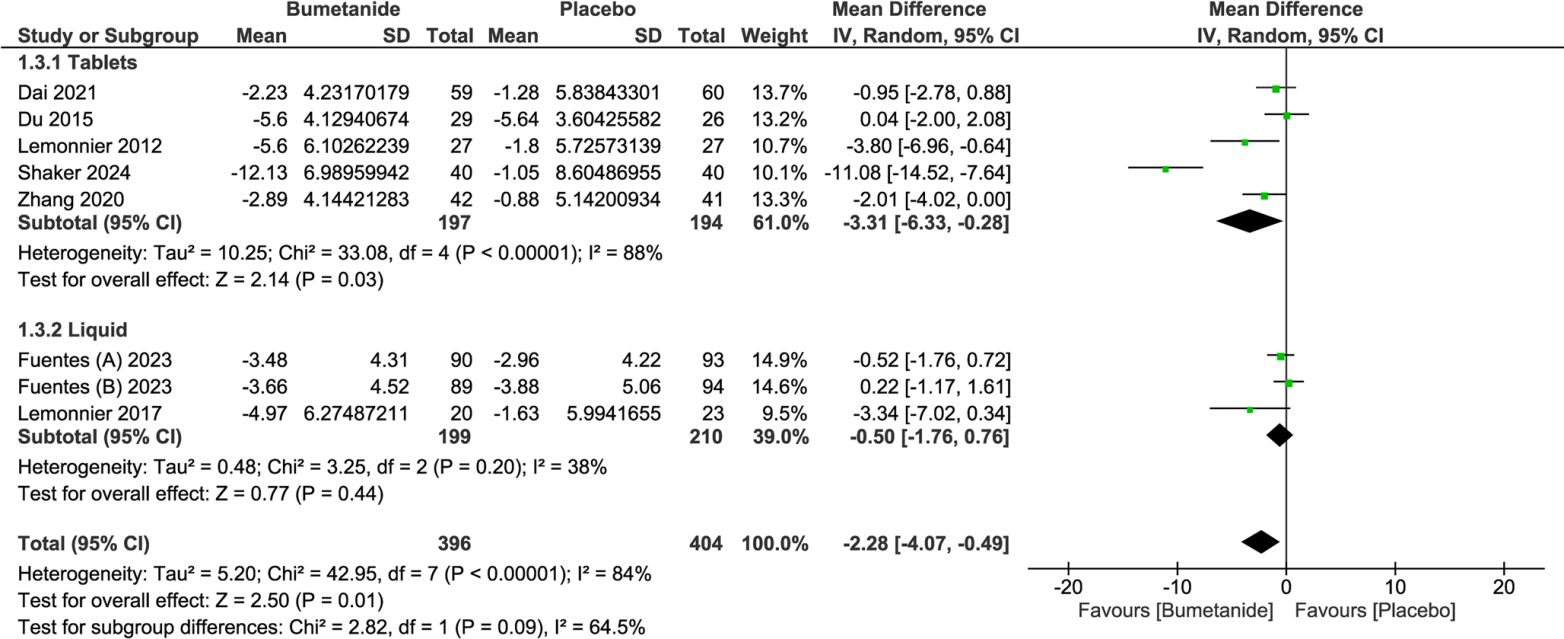
Forest plot and subgroup analysis of CARS total score based on the dosage form of the intervention

Moreover, when we compared different dosage forms, we found that bumetanide tablets, unlike liquid solution, led to a significant improvement in CARS total score when compared to placebo. However, the test for subgroup differences didn’t reach statistical significance. (Fig. 5)

### 3.4. Social responsiveness scale (SRS-2)

**Fig. 6:**
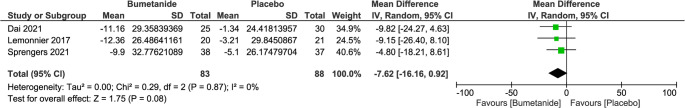
Forest plot of social responsiveness scale-2 (SRS-2) total score

The pooled analysis revealed a tendency towards enhanced social responsiveness in the bumetanide group compared to the placebo group. However, the results could not reach statistical significance (MD = −7.62; 95% CI = [−16.16, 0.92], *p* = 0.06). No heterogeneity was observed among the included studies (I2 = 0%, *p* = 0.87). (Fig. 6)

We could not include the SRS-2 data from Fuentes 2023 due to the difference in reported measures. However, they also found a non-significant difference, which aligns with the direction of our pooled estimate.

### 3.5. Clinical global impressions - efficacy index (CGI-EI)

**Fig. 7:**
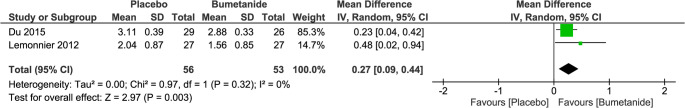
Forest plot of clinical global impression-efficacy index (CGI-EI)

The pooled analysis showed a statistically significant efficacy benefit of bumetanide compared to placebo (MD = 0.27; 95% CI = [0.09, 0.44], *p* = 0.003). There was no heterogeneity observed among the included studies (I² = 0%, *p* = 0.32), suggesting consistent results across the two studies. (Fig. 7)

### 3.6. Social interaction and communication (SI)

The impact of bumetanide on SI, using multiple scales such as ADOS social and communication sub-domains, SRS social interaction sub-domains, and the autism behavioral checklist (ABC) interaction sub-domains, revealed inconsistent results across three studies.

Subgroup analysis based on the assessment method revealed that the ADOS social and communication subscale in Dai 2021 study exhibited no statistically significant difference (SMD = 0.17; 95% CI = [−0.19, 0.53], *P* = 0.36) [15]. The pooled effect estimates of the SRS social interaction subdomain for both Dai 2021 and Lemonnier 2017 studies revealed a statistically significant difference in favor of bumetanide (SMD = −0.42; 95% CI = [−0.82, −0.01], *p* = 0.04). The autism behavioral checklist (ABC) interaction sub-domain in Du 2015 study showed no statistically significant difference (SMD = −0.19; 95% CI = [−0.72, 0.34], *p* = 0.49). (Figure S1)

Since Dai 2021 was the only study to report the social interaction domain using multiple scales: AODS and SRS, we performed various scenarios of analysis and included only one of them at a time. The data were pooled with those of other studies to obtain an overall effect estimate of the effect of bumetanide on social interaction in autistic patients.

**Fig. 8:**
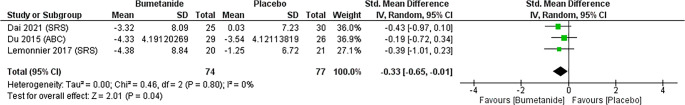
Pooled analysis of the social interaction domain of different scales (with the SRS scale of Dai et al.)

The SRS SI results imply that bumetanide may improve SI in children with autism, but the overall findings across several measures do not consistently support a significant effect. When pooling the social interaction subdomain of SRS in Dai 2021 with data from other studies, we found a statistically significant difference in favor of bumetanide (SMD = −0.33; 95% CI = [−0.65, −0.01]. *p* = 0.04). There was no heterogeneity among the included studies, which indicates consistent results across the included studies (I2 = 0%, *p* = 0.8). (Fig. 8) On the other hand, when pooling the social interaction subdomain of ADOS in Dai 2021 with data from other studies, we found no statistically significant difference between both groups (SMD = −0.06; 95% CI = [−0.40, 0.27], *p* = 0.71). There was no significant heterogeneity among the included studies (I2 = 29%, *p* = 0.24). (Figure S2)

### 3.7. Repetitive behavior and restricted interest (RRB)

The investigation of RRB across various studies using different tools, including the ADOS repetition subscale, SRS restricted interest and repetitive behavior, Autism Behavior Checklist (ABC) body and object use subscale, and RBS-R, yielded inconclusive findings. None of the reported assessment scales showed statistically significant results. The overall effects were as follows: (SMD = 0.18; 95% CI = [−0.18, 0.54], *p* = 0.31), (SMD = −0.38; 95% CI = [−0.79, 0.02], *p* = 0.07), (SMD = −0.39; 95% CI = [−0.93, 0.14], *p* = 0.15), and (SMD = −0.25; 95% CI = [−0.54, 0.03], *p* = 0.08) for ADOS, SRS, ABC, and RBS-R respectively. (Figure S3)

**Fig. 9:**
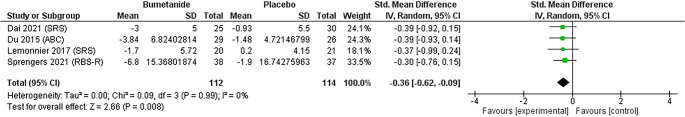
Pooled analysis of repetitive behavior and restricted interest domain of different scales (with the SRS scale of Dai et al.)

When pooling the RRB subdomain of SRS or RBS-R in Dai 2021 with the data of other studies, we found a statistically significant difference in favor of bumetanide. The Overall effect estimate was (SMD = −0.36; 95% CI = [−0.62, −0.09], *P* = 0.008) and (SMD = −0.30; 95% CI = [−0.53, −0.06], *p* = 0.01) respectively. (Fig. 9, S4) There was no heterogeneity among the included studies in both scenarios. On the other hand, when pooling the RRB subdomain of ADOS in Dai 2021 with the data of other studies, we found no statistically significant difference between both groups (SMD = −0.17; 95% CI = [−0.48, 0.14], *p* = 0.28). There was non-significant heterogeneity among included studies (I2 = 40%, *p* = 0.17). (Fig. S5)

### 3.8. Sensory affection

**Fig. 10:**
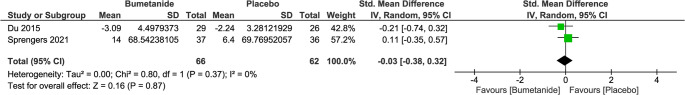
Forest plot of the sensory affection domain of different scales

Pooled analysis of two trials showed no significant improvement in sensory affection with bumetanide compared to placebo. The overall effect estimate was (MD = −0.03; 95% CI = −0.38, 0.32, *p* = 0.87). The included studies showed no heterogeneity (I² = 0%, *p* = 0.37). (Fig. 10)

### 3.9. Adverse events

Several bumetanide trials reported some common adverse events related to the intervention. Most of these adverse events were not severe and could be managed by certain interventions. Polyuria was the most commonly reported adverse event. In Dai et al., they reported that the occurrence of polyuria didn’t have a significant effect on the CARS total score [17]. Moreover, Zhang et al. reported that polyuria occurred only within 3 h of bumetanide administration and required no further management [6]. On the other hand, Fuentes et al. reported a significantly higher rate of polyuria in the bumetanide group compared to placebo [8]. Moreover, Du et al. and Lemonnier 2017 et al. reported that polyuria caused one participant to quit [14, 15]. Hypokalemia was another frequent adverse event but was usually mild and reversed with potassium supplementation or a potassium-rich diet [8],]. Other adverse events were also reported, such as loss of appetite, constipation, nausea, hyperuricemia, orthostatic hypotension, and fatigue. Serious adverse effects (SAEs) were uncommon and probably unrelated to bumetanide treatment. The bumetanide group had more study withdrawals due to adverse events [8, 14, 15].

Lemonnier 2017 suggested that the occurrence of adverse events follows a dose-dependent pattern. They reported that the rate of adverse events increased in higher dose groups, with the maximum adverse events reported in the 2 mg group. However, this pattern could not apply to efficacy, meaning that higher doses achieved better efficacy outcomes [15]. Thus, considering the tolerability and compliance, the selection of the appropriate dose is critical to achieve the desired efficacy while minimizing the adverse events.

### 3.10. Sensitivity analysis

Leave-one-out analysis was performed to assess the impact of individual studies on the overall effect. Removal of each study did not change the direction of the overall effect. In another scenario, we excluded the high-risk-of-bias studies, but the overall effect remained the same.

## 4. Discussion

### 4.1. Significance of the study

This updated meta-analysis reassessed bumetanide’s efficacy and safety in treating children with ASD by including three additional recent RCTs with larger sample sizes and longer treatment durations [8, 18]. This addition raised the total to nine eligible RCTs involving 920 participants, which is almost double the sample size of the previous meta-analysis. Additionally, two of those recent studies are large phase III multicenter trials that failed to show significant differences between the bumetanide and placebo groups [8]. These conflicting results with the previous meta-analysis mean that adding such studies to the pooled evidence is essential to obtain better insights on the efficacy of Bumetanide in ASD.

### 4.2. Summary of the findings

Our pooled analysis showed a significant difference between the bumetanide and placebo groups in the Childhood Autism Rating Scale (CARS), favoring the bumetanide group. Our pooled mean difference did not reach the − 4.5 point of minimal clinically important difference [19]. Moreover, both studies conducted by Du et al. and Lemonnier 2012 et al., with no significant heterogeneity, found that bumetanide led to a statistically significant improvement in the Clinical Global Impressions-Efficacy Index (CGI-EI) [13, 14]. On the other hand, bumetanide has no significant effect on reducing RRB after using different tools, including ADOS RRB, SRS-2 RRB, ABC RRB, and RBS-R. Moreover, no differences were observed in other outcomes, such as the Social Responsiveness Scale (SRS-2), Social Interaction and Communication (SI), and Sensory Affection.

The results of Shaker et al. were always more significant and did not overlap with the other studies. However, sensitivity analysis showed that its removal only eliminates heterogeneity without affecting the overall significance. This means that the significance of our results was not driven by a single study.

The main side effects were polyuria and hypokalemia, which, despite causing more withdrawals in the bumetanide group, were managed with dietary adjustments and potassium supplements. Other serious side effects were rare and probably unrelated to the treatment.

### 4.3. Explanation of the findings

Bumetanide is a loop diuretic and selective inhibitor of the Na-K-Cl cotransporter (NKCC1). It can improve ASD symptoms by reducing the intraneuronal chloride and transforming excitatory GABAergic signaling into inhibitory. This restores the GABAergic excitatory-inhibitory balance, which is thought to be the main contributor to ASD symptoms [6, 20] (mollajani, Zhang 2020). Our meta-analysis showed a significant change in CARS score in favor of the bumetanide group. This could be explained by the reduced GABA/Glx ratio in the insular cortex, which is believed to be responsible for sensory integration, emotional and autonomic signals, cognitive resources allocation, and behavior control [21]. Our finding was consistent with most of the included studies except for Fuentes A and B studies, where the CARS score declined post-intervention but did not reach statistical significance. This could be due to the higher baseline symptom severity of the patients included in these trials. Moreover, there was a relatively higher placebo response in these trials, which could be explained by natural fluctuations in the disease course leading to regression of symptom severity after a peak severity that was present at inclusion. Another possible explanation is that in these trials, unlike similar clinical trials, the included patients were allowed to take additional psychotropic medications, which could interfere with the accuracy of the results [8]. It’s worth noting that these studies were of a high risk of bias, which could've resulted in bias in the outcome assessment. On the other hand, among the trials that reported consistent results to our meta-analysis, Dai et al. included children below 7 years, which could lead to more favorable outcomes due to the rapid brain development and the highest rate of social and cognitive development [17]. Additionally, in Du et al., the intervention used was a combination of bumetanide and applied behavior analysis (ABA). This combination therapy was suggested to provide better results due to the biological cellular stability achieved by bumetanide, which was hypothesized to pave the way for a better brain susceptibility to ABA [14].

When conducting subgroup analysis to assess different factors that might affect the bumetanide response, we found that a significant effect of bumetanide was observed only after 3 months. There is a debate that 3 months is too short to detect treatment effect. Early trials suggested clinical signs of response, but investigators consistently urged additional research with longer duration. However, the recent phase III study (Fuentes at al.) that extended to 6 months over a large sample size failed to replicate the positive findings from earlier trials [8]. Qualitative exit interviews indicated some perceived improvement at week 26, such as emotional calmness and interaction, which may not have been fully captured by standard scales used in the trial [22]. This discrepancy could be driven by the difference in the phase of included trials. Moreover, it could be due to the nature of the treatment being symptomatic, not curative. It is worth noting that the 6-month subgroup only included 3 studies, which is too small to detect significance. Additionally, two of these studies [8] had a high placebo response, which might contribute to the overall non-significant results.

Regarding the SRS-2 scale, two out of three clinical trials reported a significant difference in favor of bumetanide. However, our pooled analysis showed non-significant differences. This could be due to the small number of trials reporting this outcome. Additionally, in Sprengers 2021 which showed non-superiority of bumetanide, there was a high placebo effect, which could be the reason why they failed to detect statistical significance [16]. This could be due to the inclusion of older children (>7 years), unlike the other two trials, which included younger age groups [15, 17].

The conflicting results among different scales could be due to the lack of standardization regarding the tool used to assess the treatment effect in ASD trials. Multiple clinical trials used CARS as the primary outcome measure. However, CARS is generally used as a screening tool for autistic symptoms, and its ability to accurately detect changes in symptoms and follow-up is doubtful. On the other hand, the SRS-2 scale is thought to be more reliable in capturing the change in symptom severity over time [23]. However, there is a lack of universally accepted measures and standardization for the assessment tools in ASD trials, which has led to conflicting and inconclusive results.

There was a great variability in the assessment tool used for social interaction and repetitive behavior. There were not enough studies in each subgroup to compare different tools. However, pooled analysis of the different scales using standardized mean differences showed a statistically significant difference. However, when replacing the SRS-2 values in Dai et al. with the ADOS values, the results became non-significant. This could be attributed to the core differences between the two scales. SRS-2 is completed by caregivers and assesses the severity of social impairment related to ASD. It provides a quantitative measure of social behaviors across various contexts, which reflects on the retrospective observations of the child’s social interaction over a certain period. On the other hand, the ADOS scale is a semi-structured observational tool administered by clinicians to obtain direct observation about the child’s social interaction, communication, and other social behaviors related to ASD [24, 25]. This difference could lead to variations in the aspects captured by each scale. Additionally, since the ADOS scale relies on current observations rather than retrospective reporting, it could be more sensitive in detecting immediate changes. However, the retrospective nature of SRS-2 provides valuable insights into the consistent behavioral patterns, which could be more useful in the clinical context.

### 4.4. Comparison with previous studies

Our meta-analysis addresses several key limitations found in the existing meta-analysis conducted by Wang et al. [7], providing a more accurate and reliable assessment of the treatment effects of bumetanide in children with ASD.

#### 4.4.1. Including recent large RCTs

Our meta-analysis covers three recent RCTs, including two major phase III multicenter studies by Fuentes et al., with a total sample size of over 400 patients, which is approximately equal to the total sample size of Wang’s study. These trials were terminated because they failed to show significant differences between the bumetanide-treated and placebo groups, highlighting the need for a more comprehensive analysis [8]. The inclusion of these trials could potentially affect the overall results.

#### 4.4.2. Methodological flaws in the previous Meta-Analysis

- **Duplicated samples in the same analysis**
  One major limitation of Wang et al.‘s meta-analysis is that they combined different subscales measuring social interaction from the same study into a single analysis. This approach has assigned disproportionate weight t certain studies, leading to inflated significance of results that might not hold if analyzed separately (1). Our meta-analysis addresses this limitation and obtains more accurate results by avoiding the inclusion of multiple scales addressing the same domain from a single study and choosing only one scale per investigation to avoid double-counting. Indeed, this approach showed varied significance from the previous meta-analysis. Moreover, it highlights the impact of scale selection on the meta-analysis outcomes due to possible differences in scope, sensitivity, specificity between tests.

- **Use** **of post-intervention values**
  Wang et al. used post-intervention values only, not MD, which do not account for baseline differences and provides less accurate estimation of the treatment effect compared to using the change values which provides more powerful comparison as it eliminates the factor of inter-person variability from the analysis [26]. In contrast, our meta-analysis employed MD and showed no significant difference from the baseline, highlighting the potential overestimation of treatment effects in the previous analysis.
- **Fixed effect model vs. random effect model**
  The included studies had heterogeneous populations of different ages, dosages, races, and severity of disease. Moreover, some studies included participants with Asperger syndrome, while others did not. To address this variability, we used a random effects model for more reliable and generalizable results, unlike Wang and his colleagues who used a fixed effects model [27].

##### 4.5. Strengths and limitations

This meta-analysis provides updated evidence regarding the use of bumetanide for ASD, highlighting the individual variations and factors that might affect treatment response. However, this study is not devoid of limitations.

One of the primary limitations is the heterogeneity and the lack of standardized methodology and assessment among the included RCTs. Variations in sample sizes, treatment durations, dosage regimens, and outcome measures, assessed by various scales and tools, complicate the direct comparison across trials. This variability may overestimate or underestimate bumetanide’s efficacy and affect the robustness and generalizability of the results. Additionally, several included studies had relatively small sample sizes, which could lead to imprecise results and potentially biased findings. Moreover, there were some variations in the inclusion criteria among the clinical trials. There was a lack of analysis of a certain subset of patients, such as those with associated ADHD or certain symptom predominance. Therefore, the results may not reflect the general ASD population and do not provide enough evidence regarding the individualized treatment for ASD patients. Although we conducted subgroup analyses for certain scales, we were limited by the sample sizes; therefore, some potentially significant subgroup effects may not have been fully explored. Moreover, several trials included in this meta-analysis had short follow-up durations, limiting the ability to evaluate the long-term effectiveness and safety of bumetanide, which is crucial given the chronic nature of ASD.

The diuretic properties of bumetanide present a challenge to effective blinding. Side effects such as polyuria, reported commonly or exclusively in the treatment arms of several trials, including Dai 2021, Shaker 2024, Zhang 2020, and Lemonnier 2017 might unintentionally reveal treatment allocation to participants or caregivers. Besides, none of the trials formally assessed blinding success, thus increasing the possibility of observer bias, particularly in caregiver-reported measures such as SRS-2. In contrast, outcomes based on structured observation, such as the ADOS, showed no significant effect. This could explain the difference in significance in social interaction and repetitive behavior when using SRS-2 vs. ADOS.

Although some studies used placebo tablets or liquid formulations matched in color, taste, timing, and administration, none employed active placebos (e.g., low-dose diuretics) to mask side effects, possibly due to ethical considerations. Future studies should consider using active placebos and formally evaluating blinding to minimize bias.

##### 4.6. Clinical implications

Although we found a significant difference in favor of bumetanide in CARS and SRS-2, the obtained effect estimate doesn’t provide clinical significance. For instance, the minimal clinically important difference (MCID) in CARS scale and SRS-2 are 4.5 and 10 points, respectively, which is slightly higher than what we reported [19, 28]. Although this MCID was included in our confidence interval, the small sample size and number of trials limit the precision of the results. Moreover, the MCID of SRS-2 was not based on established criteria but rather expert observations [28].

## 5. Conclusion

While some scales showed statistically significant differences favoring bumetanide, the effect sizes were small and inconsistent across outcomes, suggesting that bumetanide’s clinical superiority remains unproven. Larger, multicenter RCTs with standardized outcome measures and longer follow-up periods could provide deeper insight into the characteristics and evolution of ASD treatments. In addition, identifying biomarkers for treatment responders through the utilization of advanced analytical techniques may enhance the precision and applicability of bumetanide treatment of ASD patients.

## Supplementary Information

Below is the link to the electronic supplementary material.

Supplementary Material 1

## Data Availability

All data will be available from the first or corresponding author upon reasonable request.
