# Supplementary material for: The Efficacy and Safety of Bumetanide in Children with Autism Spectrum Disorder: An Updated Meta-analysis
Source: Eur Child Adolesc Psychiatry. 2025 Oct 30;35(2):413–25. doi: 10.1007/s00787-025-02890-8 (PMC12957060; doi:10.1007/s00787-025-02890-8)

**
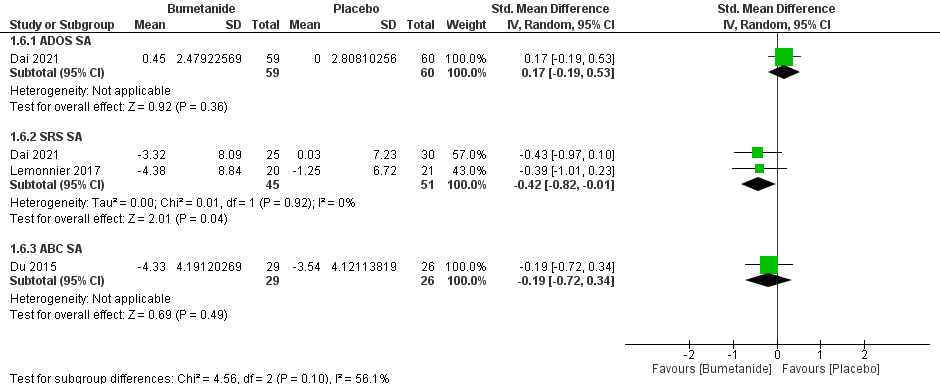
Figure S1:** Social Interaction sub-grouped based on the assessment tool

**Figure S2:** Pooled analysis of social interaction using different scales (with ADOS scale of Dai et al)


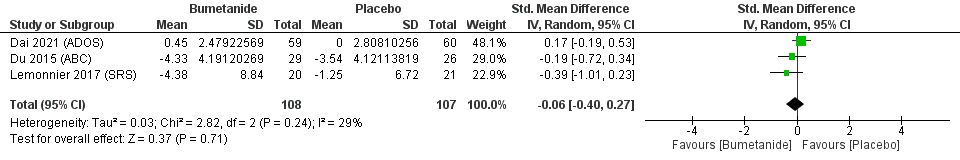


**Figure S3:** Repetitive Behavior and Restricted interest sub-grouped based on the assessment Scale


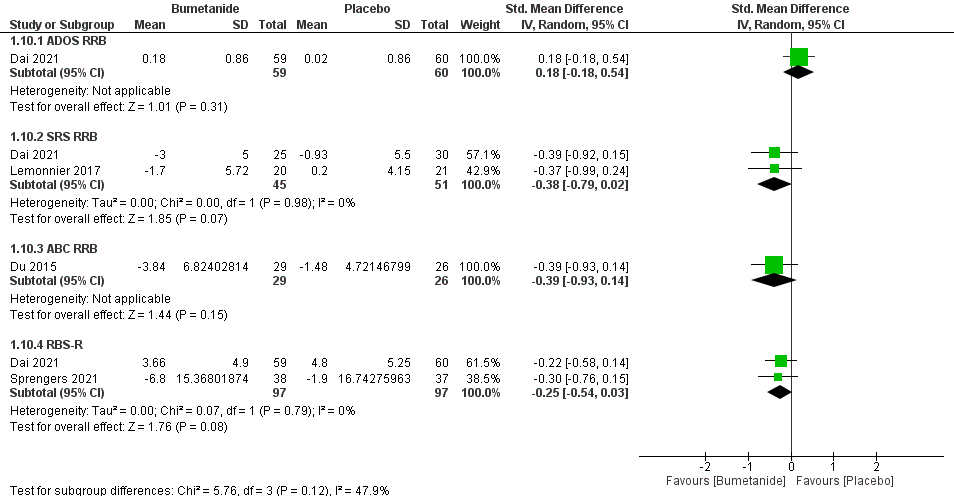


**Figure S4:** Pooled analysis of Repetitive Behavior and Restricted interest using different scales (with RBS scale of Dai et al)


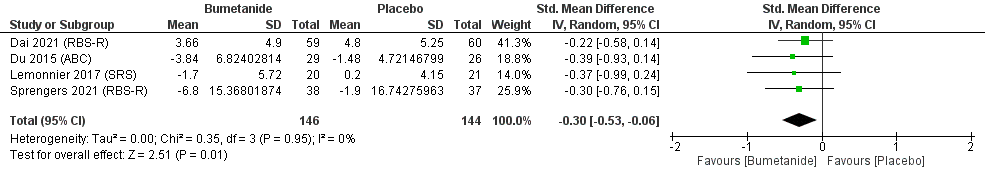


**Figure S5:** Pooled analysis of Repetitive Behavior and Restricted interest using different scales (with ADOS scale of Dai et al)


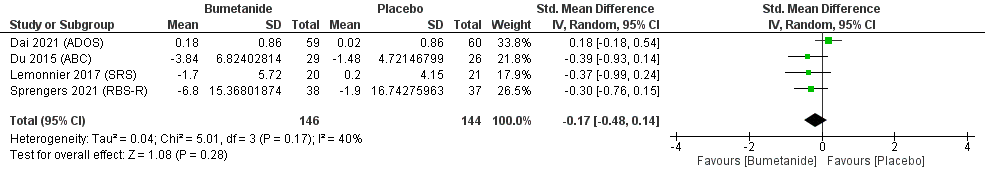

Supplement: Supplementary file 1 — Supplementary Material 1 [file 787_2025_2890_MOESM1_ESM.docx]
